# Clinical experience using trofinetide in Rett syndrome and related MECP2 diagnosis at the children’s hospital of Philadelphia post approval

**DOI:** 10.3389/fneur.2026.1776146

**Published:** 2026-04-23

**Authors:** Erin O’Connor Prange, Dennis Fleysh, Keerthana Reddy, Holly Dubbs, Eric D. Marsh

**Affiliations:** 1 Division of Neurology, Children’s Hospital of Philadelphia, Philadelphia, PA, United States; 2 Departments of Neurology and Pediatrics, University of Pennsylvania Perelman School of Medicine, Philadelphia, PA, United States

**Keywords:** caregiver reported improvements, MECP2-related neurodevelopmental disorder, Rett syndrome (RTT), RSBQ, trofinetide (Daybue)

## Abstract

**Introduction:**

Rett syndrome (RTT) is a rare neurodevelopmental disorder primarily caused by pathogenic variants in the MECP2 gene. Trofinetide (Daybue) became the first FDA-approved medication for RTT in March 2023. This study evaluates the real-world effectiveness and side effect profile of Trofinetide in 55 individuals (50 females, 5 males) with RTT or *MECP2*-related neurodevelopmental disorders over a 12-month period.

**Methods:**

Data was collected through clinic assessments and caregiver reports.

**Results:**

Results demonstrated that 75.9% of individuals experienced some improvement in RTT symptoms by caregiver report, particularly in engagement, communication, and motor skills. The side effect profile was better than the phase 3 trials with only 48.1% reporting diarrhea and 16.7% experiencing vomiting.

**Discussion:**

Overall, the findings support the effectiveness of trofinetide in the RTT population and suggests potential effectiveness in the broader MECP2 population including males and those with atypical presentations. The data highlights the need for further work to determine long-term benefits in the full spectrum of MECP2 related disorders. Finally, the study highlights the importance of titration, individualized dosing and side effect management to improve retention and outcomes.

## Introduction

Rett syndrome is a rare neurodevelopmental disorder characterized by an initial period of normal development followed by a regression in acquired skills, loss of spoken language, diminished hand function, gait abnormalities, and repetitive hand stereotypies (1, 2). This X-linked genetic disorder is primarily caused by pathogenic variants in the methyl-CpG-binding protein 2 (*MECP2*) gene and affects approximately 1 in 10,000 females and 0.2 in 10,000 males annually (3, 4). Symptoms of Rett Syndrome are diverse and include breathing dysregulation, bruxism, impaired sleep patterns, abnormal muscle tone, peripheral vasomotor disturbance, scoliosis/kyphosis, growth retardation, small cold hands/feet, inappropriate laughing/screaming spells, diminished response to pain, and intense eye “pointing” communication (1, 2). While many individuals with pathogenic variants in *MECP2* receive a clinical diagnosis of Rett syndrome (RTT), those not meeting criteria are diagnosed with atypical Rett syndrome or *MECP2*-related neurodevelopmental disorder. The phenotype in males is broad, ranging from severe neonatal encephalopathy to intellectual disability (ID), and other features commonly seen in RTT are inconsistently present (3). There has been some genotype–phenotype correlation established (5–7).

In March of 2023, Trofinetide (Daybue) became the first FDA approved medication to manage symptoms associated with RTT (8–10) after efficacy and safety was demonstrated in LAVENDER and LILAC clinical trials. In these trials, only females with a clinical diagnosis of RTT and a pathogenic or likely pathogenic *MECP2* variant were included. Both trials showed statistically significant improvements in the trofinetide treated group over placebo using the caregiver assessment Rett Syndrome Behavior Questionnaire (RSBQ) total score and the clinician assessment Clinical Global Impression-Improvement (CGI-I) score (8–10). Diarrhea and vomiting were the two prominent side effects that emerged from the trial data, but overall, it was shown to be safe. The long-term open label study data from LILAC-2 reported increased improvements over time and lower discontinuation rate (9, 10).

Not surprisingly, with the relatively recent FDA approval, information on trofinetide outside of the clinical trials is lacking. One recent publication in early 2026 of the Phase 4 “LOTUS study” was performed by Acadia Pharmaceuticals (11). They enrolled individuals who were prescribed trofinetide by their doctor and followed the participants for 1 year after initiation of trofinetide by phone or computer-based surveys of three outcome measures, the QI Disability questionnaire, the Behavioral Improvement Questionnaire, and the GI Health Questionnaire. They reported behavioral improvements in socialization, communication, and alertness domains with associated changes in global quality of life. The study was all done remotely without direct observation of any participant by the researchers (11).

As our center began to prescribe trofinetide, we aimed to examine whether clinical trial efficacy would generalize to real-world practice, extend to MECP2-related disorders beyond Rett syndrome, vary across demographic subgroups, and improve with structured caregiver education and individualized titration to the recommended maintenance dose, particularly in light of previously reported gastrointestinal adverse effects as reported in the LAVENDER and LILAC trials. We also sought to identify potential benefits of trofinetide not captured by the RSBQ by incorporating open-ended caregiver reports. This approach was intended to detect clinically meaningful changes while minimizing caregiver burden and enabling prescribers without formal training in standardized trial instruments to monitor treatment response. We report our findings after prescribing trofinetide for over 50 individuals in the first year after approval with 3–6 months of follow up for all.

## Methods

When FDA approval was obtained, all individuals with an identified *MECP2* variant known to our clinic received an email to contact our office if they were interested in an appointment to discuss treatment with trofinetide. Appointments were offered in person and via telehealth. One year after FDA approval of trofinetide, we performed a chart review of all our individuals for whom we prescribed trofinetide (and excluded those who were in the clinical trials) which included both females and males with a clinical diagnosis of typical Rett Syndrome, atypical Rett Syndrome or *MECP2-*related neurodevelopmental disorder with a pathogenic or likely pathogenic *MECP2* variant. Data was collected using an IRB exemption for existing clinical data and for quality improvement purposes.

As part of our clinical practice, we assigned all individuals a baseline Clinical Global Impression-Severity (CGI-S) score (12), and caregivers were asked to complete the RSBQ (13). While these measures were originally validated for RTT, we believe they are mostly valid in non-RTT MECP2 variant individuals as symptoms in the broader phenotype overlaps with typical RTT. Further, as these measures have some validity, we used them as relatively objective anchors to which we could compare to the more subjective caregiver reports of improvement.

Discussion to start full dose verses titration to recommended maintenance dosage occurred with each family in shared joint decision-making model. We created a titration schedule of trofinetide on an individualized basis and adjusted as needed if side effects were reported. Upon initiation of trofinetide, we requested individuals follow up in person or via telehealth for office visits at 3 and 6 months at which time we repeated CGI-S and RSBQ and scored CGI-I to objectively monitor for improvements. RSBQ completion and routine follow-up visits were encouraged but not mandated for refills, as is our standard clinical practice. As part of our routine clinical care, we documented caregiver reports on improvements and side effects discussed via phone, email and follow-up visit.

At each visit, caregivers were asked if they thought trofinetide was improving any aspect of their child’s life and if so, to describe those improvements. All caregivers were also asked to describe any adverse effects of trofinetide. If there were no significant side effects, treatment for a minimum of 6 months was encouraged to assess if the individual would experience improvements, but joint decision-making model was implemented, and families could decide at any time to stop trofinetide. If side effects were not manageable, for example persistent vomiting despite interventions, clinicians recommended trofinetide be stopped.

At the baseline visit to discuss trofinetide initiation, a handout was provided to all families with recommended dosage for weight, titration if appropriate, administration recommendations, diarrhea management strategies, and follow up expectations. Diarrhea management instructions for individuals started on full recommended dose included stopping all constipation medication (such as polyethylene glycol, milk of magnesia, and sennoside) and starting fiber supplementation 2 days prior to initiation of trofinetide. Individuals who started with titration that was less than 50% of the goal dosage were instructed to stop constipation medication and start fiber supplements when stools became more frequent and/or looser than baseline. Families were instructed to increase fibrous foods and add bananas into diet if possible. Fiber supplement instructions were to start with 1 teaspoon per day of psyllium and increase as needed and tolerated to a maximum of 1 tablespoon twice daily. Both groups were instructed to restart polyethylene glycol if the individual did not have a bowel movement within 48 h. Both groups were also told that trofinetide dose could be decreased or temporarily held, and titration slowed if diarrhea occurred to allow time to increase fiber and manage stools. Both groups were also given weight-based dosages for loperamide but instructed not to use for more than 2–3 days without calling the office as our preference was to manage stools with titration and fiber rather than long term use of loperamide. Caregivers were asked to email via secure electronic medical record portal if diarrhea, vomiting or other side effects occurred to discuss adjustment in trofinetide dosing/titration, increase fiber, change to alternative fiber source, and/or other interventions in attempt to mitigate side effects prior to stopping trofinetide.

In the chart extraction, we collected age, *MECP2* variant, whether dose was titrated, percentage of goal dose reached, side effects, CGI-I, CGI-S, RSBQ, concurrent medications, and all qualitative comments about individual experience on trofinetide. Qualitative comments about each individual’s treatment experience were reviewed by a clinician and grouped either as improvements or side effects. Categories were created by treating clinicians to encompass the improvements most frequently reported to aid in statistical review. “Increased engagement” included comments such as better eye contact, more focused, more alert, and more aware of the environment. “Increased communication” encompassed comments regarding increase in vocalizations and better use of augmentative communication device such as more complex phrases, more spontaneous use, use to engage in conversation and more complex takes such as initiating a request. “Fine motor” category included comments such as calmer hands, less hand stereotypies, and any improvement in hand use such as reaching out, grasping more, holding objects longer, controlled release of objects and more purposeful use of hand. “Gross motor category” included comments regarding improvement in gait such as faster pace, steadier, ability to navigate stairs or uneven ground, and less assistance required. “Breathing dysregulation” category included comments describing a decrease in frequency or intensity of hyperventilation and/or breath holding and less cyanosis. Data on any change in seizures was also collected.

Data was entered into an excel spreadsheet and analysis of dose initiation, max dosage tolerated, duration of treatment, improvements and side effects were all summarized and statistical comparisons performed, and figures made using R and Python [Figures made using Python Software Foundation. (2021)]. Python (Version 3.10) (Computer software).^1^ RStudio Team. (2024). *RStudio: Integrated Development Environment for R (Version 2024.4.2.764)* (Computer software). Posit Software, PBC.^2^

Concurrent medications taken while also taking trofinetide are listed in a supplemental table, but no additional analysis was completed to determine if relationship exists between medications and trofinetide because reason for taking, dosage and duration were not extracted from chart upon retrospective review. This is included for informational purposes only as no serious adverse reactions were noted during this observational period when these medications were used concurrently.

## Results

### Study population

**Figure 1:**
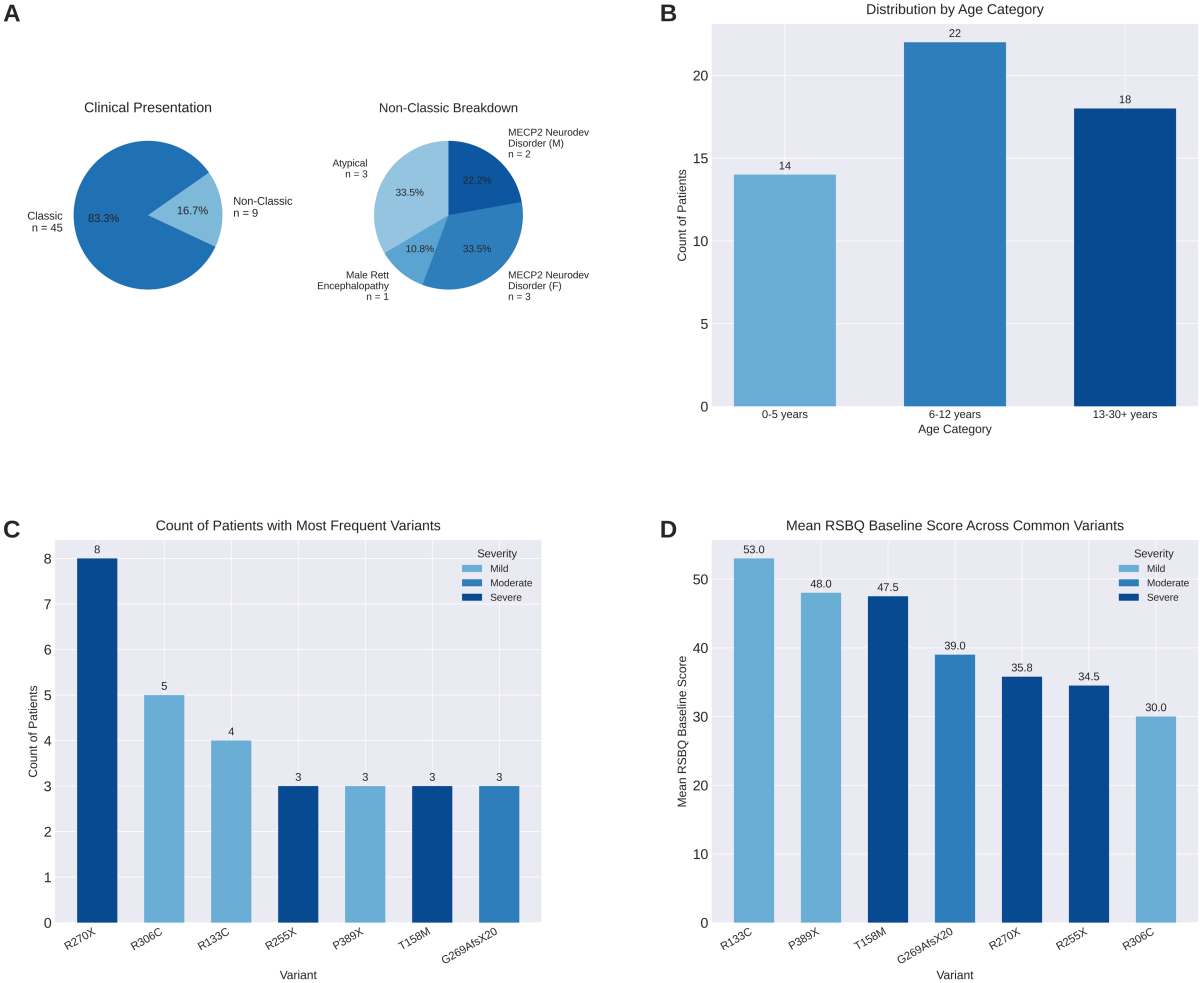
Clinical presentation profile and characteristics in cohort (*n* = 54). **(A)** Distribution of classic versus non-classic variant presentations in the study population (left, *n* = 54), and breakdown of non-classic variant subtypes (right), including atypical Rett syndrome, male Rett encephalopathy, and *MECP2*-related neurodevelopmental disorder in males (M) and females (F). **(B)**. Age distribution of individuals across three developmental categories: 0–5 years (*n* = 14), 6–12 years (*n* = 22), and 13–30 + years (*n* = 18). **(C)** Frequency distribution of most common *MECP2* variants, stratified by clinical severity (mild, moderate, severe). **(D)** Mean RSBQ (Rett Syndrome Behavior Questionnaire) baseline scores for the most frequent *MECP2* variants, grouped by severity classification. Higher scores indicate more severe behavioral symptomatology.

From April 2023 to June 2024, 55 individuals, 50 females and 5 males, ranging in age from 14 months to 38 years were started on trofinetide. For analysis, we divided the group into three age categories (Figure 1B). The cohort included 45 individuals diagnosed with typical Rett syndrome, 3 atypical Rett syndrome, 5 *MECP2*-related neurodevelopmental disorder and 2 male Rett encephalopathy (Figure 1A). Multiple variant types were represented in this cohort with the most common being R270X (*n* = 8) and G269AfsX20 (*n* = 3) (Figure 1C). Variants were categorized into three severity groups based on previously established genotype–phenotype correlations: mild, moderate, or severe (6). The RSBQ scores are tracked with severity of variant in this small cohort (Figure 1D).

One male with Rett encephalopathy was excluded in the outcome results (but not side effect patterns) as he was hospitalized for the entirety of his 12 weeks of treatment, RSBQ was not obtained, and there was no outpatient follow up. Titration and side effect management instructions were given to the inpatient care team. There was no perceived benefit, diarrhea occurred, and he experienced multiple other systemic medical co-morbidities secondary to male encephalopathy, so treatment was stopped at 12 weeks. We include him in this report as he was under 2 years of age, and no unforeseen adverse reactions occurred suggesting safety in this younger age group and need for further study.

### Efficacy

**Figure 2:**
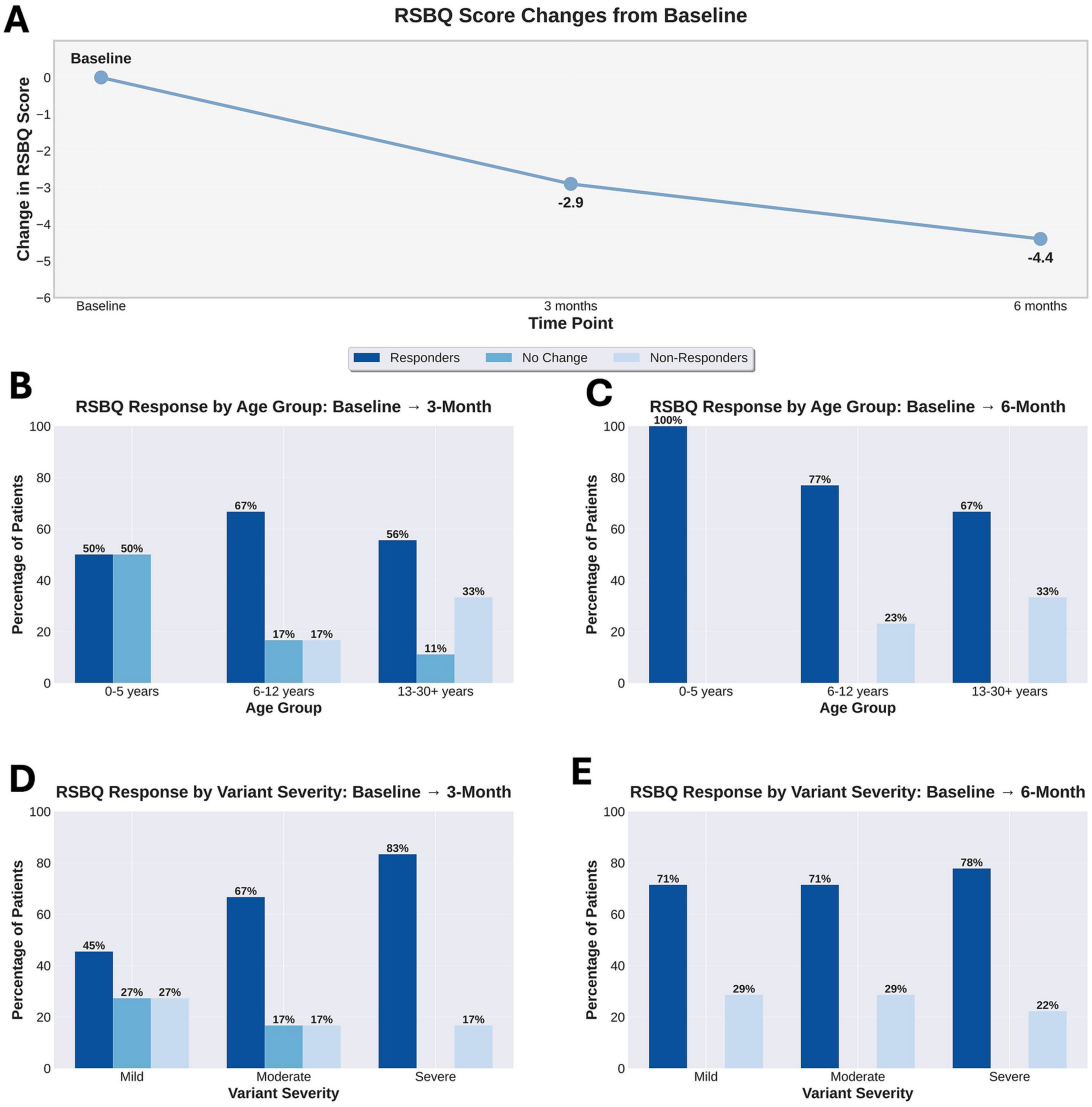
RSBQ behavior responses to trofinetide treatment across age groups and variant severity (*n* = 23). **(A)** Mean change in RSBQ (Rett Syndrome Behavior Questionnaire) scores from baseline at 3-month (*n* = 23) and 6-month (*n* = 23) follow-up timepoints. Negative values indicate improvement in behavior symptoms, with mean reductions of −2.9 points at 3 months and −4.4 points at 6 months. **(B)**. RSBQ response trajectories at 3 months stratified by age group. Percentage of individuals classified as responders (score decrease), no change, or non-responders (score increase) comparing baseline to 3-month timepoint across three developmental categories: 0–5 years (*n* = 2), 6–12 years (*n* = 12), and 13–30 + years (*n* = 9). **(C)** RSBQ response trajectories at 6 months stratified by age group. Percentage of individuals in each response category comparing baseline to 6-month timepoint across developmental categories: 0–5 years (*n* = 3), 6–12 years (*n* = 13), and 13–30 + years (*n* = 6). **(D)** RSBQ response trajectories at 3 months stratified by variant severity. Percentage of individuals in each response category comparing baseline to 3-month timepoint across severity classifications: Mild (*n* = 11), Moderate (*n* = 6), and Severe (*n* = 6). **(E)** RSBQ response trajectories at 6 months stratified by variant severity. Percentage of individuals in each response category comparing baseline to 6-month timepoint across severity classifications: Mild (*n* = 7), Moderate (*n* = 7), and Severe (*n* = 9). Overall response rate increased from 60.9% at 3 months to 73.9% at 6 months across all groups.

RSBQ was completed for 51 of 54 individuals at baseline (94.4%). Fourteen individuals (25.9%) stopped treatment before 12 weeks, so follow-up data was not obtained. At the 3 month follow up visit, RSBQ was repeated in 23 of the remaining 40 individuals (62.2%). Remainder either did not complete RSBQ or did not return as requested for the 3-month visit. Three more individuals stopped trofinetide after week 12 but before month 6. RSBQ was completed in 23 of the remaining 37 individuals (62.2%) at 6 months (Figure 2).

Of those who completed RSBQ at 3 months, 60.9% demonstrated a reduction in scores (improvement of symptoms), 17.4% demonstrated no change, and 21.7% demonstrated an increase (worsening of symptoms). By 6 months, 73.9% demonstrated a reduction in RSBQ, 0% demonstrated no change, and 26.1% demonstrated an increase. On average, individuals demonstrated a 2.9-point reduction in RSBQ scores from baseline to 3 months and a 4.4-point reduction from baseline to 6 months.

**Figure 3:**
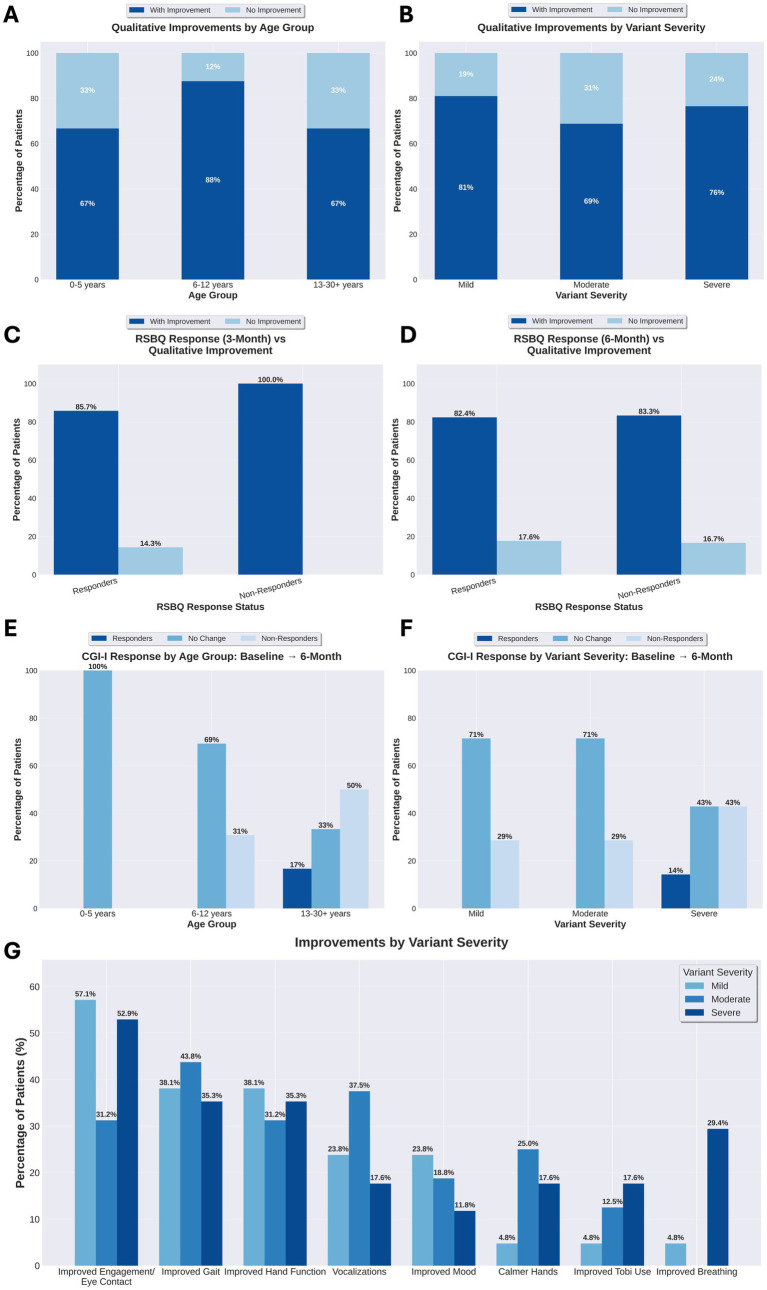
Qualitative improvements, RSBQ-qualitative concordance, CGI-I outcomes, and clinical improvement domains by age and variant severity. **(A)**. Percentage of individuals demonstrating any caregiver-observed qualitative improvement by age group (*n* = 54 total). Across all age groups, 75.9% (41/54) of individuals demonstrated at least one qualitative improvement. The 6–12 years age group showed the highest improvement rate at 87.5% (21/24), followed by 0–5 years at 66.7% (6/9) and 13–30 + years at 66.7% (14/21). **(B)**. Percentage of individuals demonstrating any caregiver-observed qualitative improvement stratified by MECP2 variant severity (*n* = 54 total). Overall, 75.9% (41/54) of individuals showed at least one qualitative improvement. By severity: Mild variants showed 81% improvement rate (17/21), Moderate variants 68.8% (11/16), and Severe variants 76.5% (13/17). **(C)**. Concordance between RSBQ response status at 3 months and presence of qualitative improvements. Among RSBQ responders (*n* = 14), 85.7% demonstrated qualitative improvements, while 14.3% showed no qualitative improvement. Among non-responders (*n* = 5), 100.0% demonstrated qualitative improvements. **(D)**. Concordance between RSBQ response status at 6 months and presence of qualitative improvements. Among RSBQ responders (*n* = 17), 82.4% demonstrated qualitative improvements, while 17.6% showed no qualitative improvement. Among non-responders (*n* = 6), 83.3% demonstrated qualitative improvements, while 16.7% showed no qualitative improvement. **(E)**. Clinical Global Impression of Improvement (CGI-I) outcomes at 6 months stratified by age group (0–5 years *n* = 2, 6–12 years *n* = 13, 13–30 + years *n* = 6). Results show 100% no change in the 0–5 years group, while the 13–30 + age group showed the highest improvement rate at 16.7%. **(F)**. CGI-I outcomes at 6 months stratified by variant severity (Mild *n* = 7, Moderate *n* = 7, Severe *n* = 7). Overall results indicate 4.8% showed improvement, 61.9% had no change, and 33.3% worsened. The severe variant group demonstrated 14.3% improvement. **(G)**. Percentage of patients demonstrating caregiver-reported improvement across eight clinical domains, grouped by MECP2 variant severity classification (Mild *n* = 21, Moderate *n* = 16, Severe *n* = 17). Combined data from 3-month and 6-month follow-up assessments (*n* = 41 of 54 patients reported improvement in at least one domain). Categories are ordered by overall frequency of improvement across all severity groups, with improved engagement/eye contact being most common (57.1% mild, 31.2% moderate, 52.9% severe), followed by improved gait and hand function.

**Figure 4:**
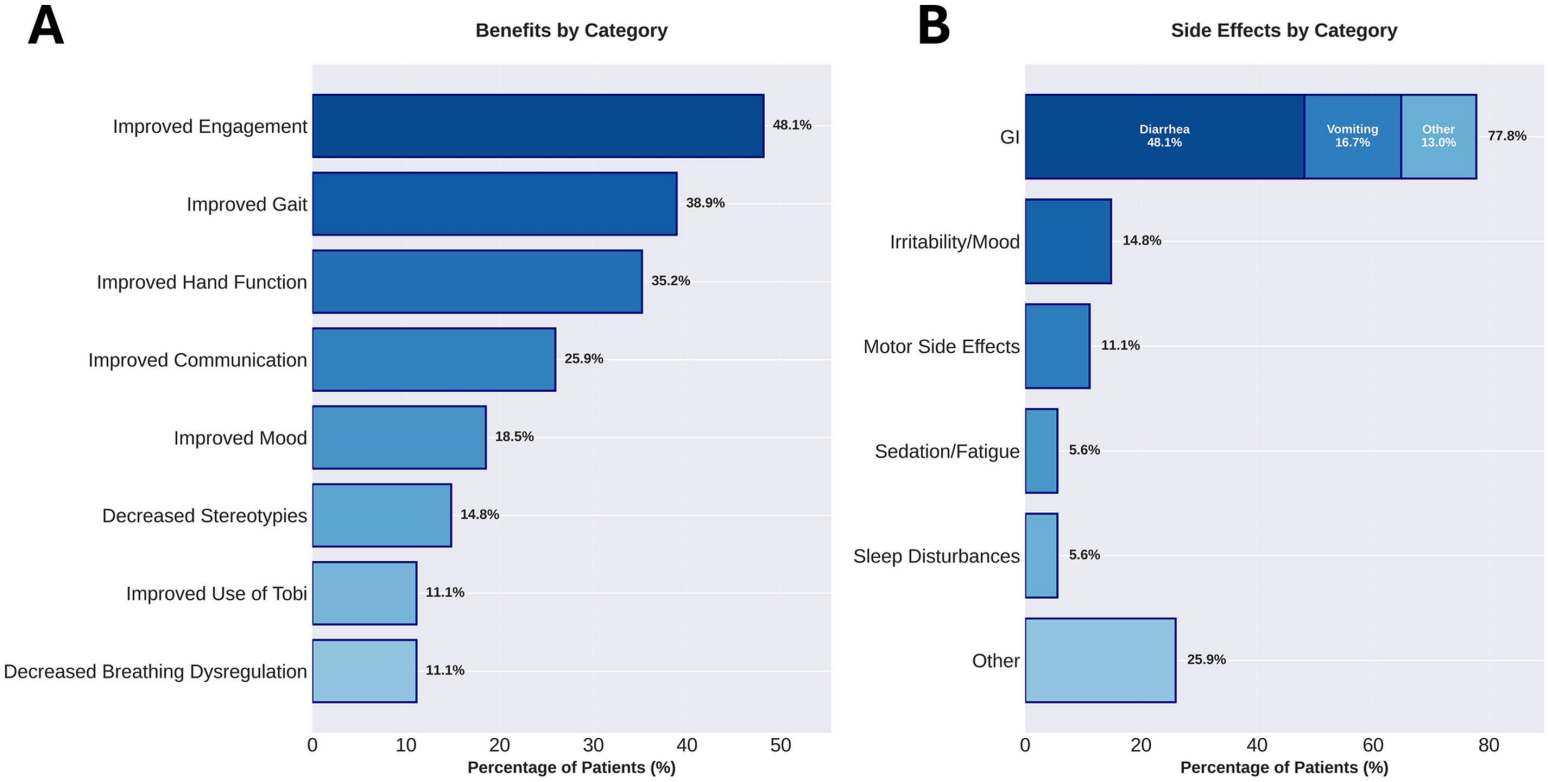
Caregiver-reported benefits and side effects of trofinetide treatment (*n* = 54). **(A)** Percentage of patients demonstrating caregiver-reported benefits across eight clinical domains. The most common improvements were enhanced engagement/eye contact (48.1%, *n* = 26), improved gait (38.9%, *n* = 21), and improved hand function (35.2%, *n* = 19). Additional benefits included improved communication (25.9%, *n* = 14), improved mood (18.5%, *n* = 10), decreased stereotypies (14.8%, *n* = 8), improved use of Tobii eye-gaze communication device (11.1%, *n* = 6), and decreased breathing dysregulation (11.1%, *n* = 6). Categories are ordered by frequency of occurrence. **(B)** Percentage of patients experiencing side effects by category. Gastrointestinal (GI) side effects were most common (77.8% overall, *n* = 42), comprising diarrhea (48.1%, *n* = 26), vomiting (16.7%, *n* = 9), and other GI symptoms (13.0%, *n* = 7). Additional side effects included irritability/mood changes (14.8%, *n* = 8), motor side effects (11.1%, *n* = 6), sedation/fatigue (5.6%, *n* = 3), sleep disturbances (5.6%, *n* = 3), and other side effects (25.9%, *n* = 14). The GI category is displayed as a stacked bar showing the breakdown of GI-related adverse events.

In addition to the RSBQ, we collected qualitative reports from every caregiver who returned for a follow up appointment by asking them to describe any perceived improvements or side effects since starting treatment with trofinetide. Analyzing these caregiver reports, we found that 41 of the 54 individuals (75.9%) experienced some type of improvement and 31 of those 41 (75.6%) experienced more than one improvement. A total of 116 improvements were reported for an average of 2.83 improvements per individual who reported benefits. (Figure 3). The most notable areas of reported improvement include increased engagement in 26/54 (48.1%) individuals, improved gait in 21/54 (38.9%) individuals, and improved hand use in 19/54 (35.1%) individuals. Other categorized improvements observed included improved communication/vocalizations (14/54, 25.9%), improved mood (10/54, 18.5%), calmer hands/reduced hand stereotypies (8/54, 14.8%), improved Tobii use (6/54, 11.1%), and decrease in breathing dysregulation (6/54, 11.1%) (Figure 4).

Of the 5 individuals with *MECP2-*related neurodevelopmental disorder, RSBQ was completed at 6 months for 3/5 (60%) patients; two individuals each decreased by 7 points and the third decreased by 18 points. Caregivers of the two individuals who did not complete follow up RSBQ both reported some qualitative improvement, but one stopped at 5 months due to perceived improvement not being large enough to continue therapy. Improvements reported across the 5 individuals included: improved hand use (3), improved gait (3), increased language (3), more attentive (2) less mouthing (1), and less anxious (1). Although a small sample size, this data demonstrated treatment could be beneficial to those with *MECP2*-related neurodevelopmental disorders, a group not included in the original trial data.

One male with Rett encephalopathy included in the results tolerated full dose without reported side effects, and caregivers listed improvements including him being calmer, less anxious, more observant, and opening hands more.

At the 3-month visit, we repeated CGI-S, assigned CGI-I, asked caregivers if they felt there was improvement, and then collected qualitative comments. Thirty-seven individuals returned for 3-month visit, 12 individuals stopped trofinetide without returning for visit, and 5 individuals continued treatment but skipped the 3-month visit. Of the 37 who returned, 29 caregivers answered yes (78.4%) that they perceived improvement and 8 (21.6%) answered no that there was no perceived improvement. For those same individuals, CGI-I was minimally improved (3) in 7 individuals (18.9%), no change (4) in 28 patients (75.7%), minimally worse (5) in one (2.7%) and much worse (6) in one (2.7%).

We next analyzed the responses by subgroup (CGI-S score or severity of *MECP2* mutation) to determine if any group responds better than any other subgroup. Responses were analyzed based on variant severity, age at treatment initiation, baseline CGI-S, and max % goal dose reached.

### Variant severity

The total reported improvements for each severity group were: 43 improvements in the mild severity group (*n* = 21), 35 improvements in the moderate group (*n* = 16), and 38 improvements in the severe group (*n* = 17). These findings suggest individuals can experience improvements regardless of mutation severity, with no statistical difference between groups [chi-square test of independence: χ^2^(2) = 4.311, *p* = 0.1158].

For the RSBQ change, one-way ANOVAs were conducted to examine whether mean improvement differed across mutation severity groups at both 3-month and 6-month follow-ups. At 3 months, the severe group showed the highest mean improvement (−4.83, SD = 9.92), followed by the mild group (−2.55, SD = 9.59) and the moderate group (−1.5, SD = 8.57); however, these differences were not statistically significant [*F*(2, 20) = 0.26, *p* = 0.773, η^2^ = 0.025]. Similarly, at 6 months, the moderate group showed the highest mean improvement (−6.14, SD = 9.35), followed by the mild group (−4.29, SD = 8.64) and the severe group (−3.00, SD = 9.14), but again, these differences did not reach statistical significance [*F*(2, 20) = 0.24, *p* = 0.791, η^2^ = 0.023]. These findings indicate that variant severity does not significantly influence the degree of RSBQ improvement at either timepoint, suggesting that individuals across all severity categories demonstrate similar patterns of behavioral improvement with treatment, regardless of their baseline mutation severity classification.

### Improvements by age

To determine if age had an impact on improvements reported, we separated individuals into 3 age groups: 0–5 years, 6–12 years, and 13 and older (Figure 1A). There were 27 improvements in the 0–5-year group, 54 improvements in the 6–12-year group, and 31 improvements in the 13 + group. The mean number of improvements per individual was highest in the 6–12 years group (3.04), followed closely by the 13 + years group (2.76), and the 0–5 years group (1.20). These results suggest that improvements can be experienced across the lifespan (Table 1).

**Table 1:** Improvements by age category.

Age category	*N*	Mean	SD	Range (min-max)
0–5 years	9	1.11	1.05	0 to 3
6–12 years	24	2.50	1.53	0 to 5
13–30 + years	21	1.90	1.87	0 to 5

Improvements reported by families by age during treatment.

### Improvements by severity score

To determine if the Rett Syndrome Severity Score impacts report of improvements or RSBQ score changes, we compared caregiver reported improvements by baseline Clinical Global Impression-Severity (CGI-S) scores in the 54 individuals (Table 2). Individuals with a CGI-S score of 2 demonstrated the highest average number of improvements (3.00) which could suggest those with mild symptoms respond most favorably to treatment but due to the small sample size this cannot be generalized without future studies and a larger data set to analyze. Individuals with moderate CGI-S scores (4 and 5) exhibited improvements close to the overall mean (2.04 and 2.75), respectively. Interestingly, individuals with CGI-S scores of 3 and 6 exhibited the lowest average improvements (1.22 and 1.43, respectively). From this small sample cohort, we cannot predict potential relationships between baseline severity and treatment response but demonstrate improvements can be seen in all severity categories.

**Table 2:** Analysis by CGI-S score (baseline).

CGI-S score	*N*	Mean /individual	SD	Range (min-max)	RSBQ baseline (mean)	RSBQ change 3 M (mean)	RSBQ change 6 M (mean)
2	3	3	1	2–4	22	1	Data not available
3	9	2.56	1.42	1–5	34.3	-7	−8
4	23	1.65	1.61	0–5	36.6	−3.3	−3.2
5	10	1.7	2	0–5	34.5	−8.5	−2.2
6	7	2.57	1.9	0–5	30.1	3.6	−1

Improvements reported in Rett Syndrome Behavior Questionnaire by baseline clinician global impression of severity score at 3 and 6 months of treatment.

To further examine the relationship between CGI-S and treatment response, the baseline Rett Syndrome Behavior Questionnaire (RSBQ) scores and their corresponding changes were analyzed (Table 3).

**Table 3:** Improvements by severity group.

Severity	*N*	Mean/individual	SD	Range (min-max)
Mild	21	1.95	1.6	0–5
Moderate	16	2	1.71	0–5
Severe	17	2.18	1.78	0–5

Improvements reported by families by baseline severity of mutation during treatment.

Individuals with CGI-S scores of 5 and 6, who had higher baseline RSBQ scores (38.8 and 35.4, respectively), exhibited moderate reductions in Rett syndrome behaviors (−4.2 and −6.3, respectively). Conversely, individuals with CGI-S scores of 3 and 4 demonstrated minimal improvements (−1.7 and −0.8, respectively) despite having relatively high baseline RSBQ scores (36.5 and 37.3, respectively).

Individuals with CGI-S score of 2 had the lowest average baseline RSBQ score (25.67) and the greatest reduction (−10.67) but the smallest sample size (*n* = 3) which prohibits generalizability. The overall mean RSBQ change across all individuals was −3.63, reflecting a general trend of symptom improvement and demonstrates those with higher RSBQ scores (38.8) as well as lower scores (25.7) can experience reductions in symptoms.

### Improvements by final dose

Sixteen individuals (16/54; 29.6%) were started on full dose trofinetide verses 38 of those 54 (70.54%) choosing to titrate to recommended maintenance dose based on weight banded dosing. Titration increments and length of time to goal dose varied with providers considering aspects such as caregiver preference, medication history, nutritional status, and ability to tolerate volume. Thirty-five individuals (35/54; 64.8%) reached full dose as recommended by weight banded dosage. The remaining 19 (35.2%) continued at maximum tolerated dose, which varied between 25 and 99% of indicated maintenance dose. When analyzing improvements by final dose category, there is a trend in which higher doses have greater caregiver reported improvements. The full-dose group exhibited a mean improvement of 2.54 improvements per individual, the 76–99% band exhibited 3.12, the 51–75% band exhibited 2.80 and the 25–50% band exhibited 2.17 improvements per individual (Table 4).

**Table 4:** Analysis by dose category.

Dose category	Total improvements	Mean/individual	SD	Range (min-max)	RSBQ drop 3 M (mean)	RSBQ drop 6 M (mean)
25–50% (1/4 dose)	6	2.17	2.23	0–6	−1.67	1
51–75% (1/2 dose)	5	2.8	0.84	2–4	−5.5	−4
76–99% (3/4 dose)	8	3.12	1.64	1–5	−1.4	1
100% (full dose)	35	2.54	1.9	0–7	−3.31	−6.87

Improvements reported by families by maximum dose of trofinetide during treatment.

Of note, the majority of individuals (16/23) in the 76–99% category was classified as having mild severity by the CGI-S (with only 4 in the moderate category and 3 in the severe category). This could partially explain the higher average improvement observed in this group, as it is not clear with the small sample size presented, if baseline severity influences the degree of response to treatment.

### Side effects and discontinuation

In our cohort, 26/55 (47.3%) reported diarrhea and 9/55 (16.4%) experienced vomiting but the majority of these individuals did not discontinue treatment as a result. We reviewed charts from all individuals who did not complete at least 6 months of trofinetide. Fourteen individuals (25.5%) stopped trofinetide before completing 12 weeks of treatment with mean time to discontinuation of 3.8 weeks (range 3 days to 10 weeks). Reasons for discontinuation included diarrhea (8 individuals, 5 of whom also had another reported side effect), vomiting (4), decline in stability (2), no benefit in addition to other reported side effects (2), refusal to drink (1), poor sleep (1), breakthrough menstrual cycle and negative mood (1).

Three individuals who stopped treatment due to vomiting tried interventions such as lowering dose of trofinetide/titrating slower, starting reflux medication, and referral to GI specialist for treatment with medication to help with GI motility without effect. Of note, these three individuals reported improvements in other areas while taking trofinetide. The fourth individual stopped after just 3 days without trying interventions to manage vomiting and had no reported benefit. There were an additional eight individuals who experienced vomiting but had resolution of vomiting with previously described interventions and were able to continue treatment.

Four individuals discontinued trofinetide after 12 weeks but before 6 months. Among these four individuals, two experienced mild improvement, while the other two reported no noticeable benefits. Reported side effects included diarrhea (3), poor sleep (2), irritability (1), refusal to drink (1), increased stereotypies (1), and dystonia (1).

When comparing those who dropped out to those who reported a benefit, a 1.53:1 benefit to side effect ratio was calculated.

## Discussion

Trofinetide is the first and currently only FDA-approved medication to treat symptoms associated with Rett Syndrome, and our retrospective real-world experience data supports the clinical trial data and extends it in several important ways. Like the clinical trial data, our real-world experience suggests that trofinetide is effective in reducing symptoms associated with RTT, and the most reported side effect is diarrhea (8, 9). In addition, our experience demonstrates potential effectiveness in non-classic Rett syndrome presentations including males and those with *MECP2*-related neurodevelopmental disorder. Second, reports of diarrhea were significantly less than the trial data suggesting caregiver education, titration, dose modifications, and close follow-up may help manage gastrointestinal side effects. Third, we demonstrate that caregivers report meaningful improvements that may not be captured by standard scales such as the RSBQ or CGI-I when evaluating efficacy outside of a clinical trial suggesting future studies to explore alternative ways to capture caregiver perceived improvements is needed and reliance on RSBQ to demonstrate effectiveness to continue therapy may not be the only measure. Lastly, although sample size is small, our exploratory data of subgroups show that improvements can be reported in any individual with a pathogenic *MECP2* variant regardless of gender, age, baseline CGI-S or mutation severity.

In the Lavender phase 2 and 3 clinical trials, there was a 5.1 drop in RSBQ between baseline and 12 weeks and 7.1-point reduction in RSBQ from 12 to 40 weeks (8, 9). Our real-world data also demonstrated greater improvement over time, but average reduction was not as robust as the trial data. We describe an average 2.9 point RSBQ reduction by 12 weeks and 4.4 point reduction by 24 weeks (8, 9). Our study is real-world clinical experience and hence, an open-label study. While traditionally, in an open-label study one would expect a greater improvement than placebo-controlled study, we did not find this. There are a few potential reasons that may explain the slightly lower RSBQ reduction. First, we included a larger range of clinical presentations, baseline CGI-S, and did not have exclusion criteria that would restrict those individuals actively going through regression or who were unstable due to other medical conditions compared to the clinical trial. Second, social media has had mixed views on the effectiveness of Trofinetide, hence caregivers may have had a preconceived impression of lack of improvements and/or side effects from experiences shared on social media. Third, outcome measure sensitivity may have also influenced our numbers as we did not limit completion of RSBQ to the same caregiver each time as was required in the clinical trials. For these reasons, we further analyzed the data and found that of those individuals with RSBQ reduction (trofinetide responders), there was average 7.1 point RSBQ reduction by 3 months and of those with RSBQ increase (non-responders), there was an average 6.6 point increase- both of which are closer to the Lavender trial results.

Regarding caregiver reported improvements, we used open-ended questions to collect qualitative comments on improvements and side effects. While a limitation of this method is introducing variability in caregiver reporting and clinician discretion in categorizing these reports, a benefit is that we were able to capture a larger range of improvements considered meaningful to caregivers. Using the clinician assessment of CGI-I at month 3, only 7 individuals of the 37 who were on treatment and returned for visit (18.9%) had CGI-I of 3 indicating minimal improvement but 29 (78.4%) answered “yes” that they perceived improvement and caregivers reported 41 of the 54 individuals (75.9%) showed some type of improvement. This difference highlights the need to take caregiver perception into account and raises questions of whether our current scales are sensitive enough to capture what is considered meaningful improvement to a caregiver and family. As caregivers report positive findings, the question about the clinical meaningfulness of these changes should be asked. As part of the Rett and Related Disorders Natural History Study, families were asked what symptoms were most problematic for their loved one with Rett syndrome (14) and our data shows that trofinetide had an impact in those areas, specifically communication and hand use. Whether the degree of improvement on trofinetide is substantial enough to warrant ongoing therapy is a question that the clinical and caregiver community should discuss further. In the setting of gene therapy, there has been a recent study to look at what is considered meaningful change (15). A similar study thinking about what would be considered meaningful for a small pharmaceutical with a strong safety profile could be performed.

Our results are similar to the recently completed Phase 4 study from Acadia Pharmaceuticals (11). The LOTUS study reports on a larger cohort, 227, than our study, but relied on on-line or phone-based surveys of caregivers reporting improvement from trofinetide using Quality of Life Inventory-Disability (QI-Disability) and Behavioral Improvement Questionnaire (BIQ). The BIQ was a new measure which essentially mimics our qualitative improvements interview, but without the direct caregiver-clinician interaction that allows for clarification and questioning of any perceived improvements. The QI-Disability, a validated measure for children aged 5–18 years with intellectual disability and validated for adults with RTT supported the improvements documented in the BIQ (11). Thus, LOTUS data and our data highlight need of caregiver input.

We also demonstrate that being flexible in the final target dose and the titration schedule could improve both the tolerability and retention of individuals. In the trials, there were fixed doses and no titration schedule. While this is to be expected in a trial, recommending titration strategies to improve tolerability is an area for future studies to consider.

In regard to side effects and dropout rate, we show 47.3% reporting diarrhea and 25.5% (14/55) dropout rate in the first 12 weeks compared to 80.6% reporting diarrhea and drop out of 24.7% (23/93) in randomized Phase 3 trial results (8). This suggests lessons learned from clinical trials which led us to creating education for caregivers to stop constipation medication, add fiber, and titrate to maintenance dose may decrease side effects such as diarrhea but do not necessarily increase retention. Interestingly, most individuals who discontinued treatment reported it was due to adverse effects and not lack of perceived benefit so further exploration of relationship between side effects and retention would be helpful. We emphasize here that caregiver education was provided by the same clinician with experience in using trofinetide and close follow up was completed throughout the initiation process which may have led to this significant decrease in reported diarrhea which may not be reproducible in all clinical settings. It does support that trofinetide appears safe and no unexpected serious adverse events emerged during this observational period.

Finally, we show that in a real-world setting, clinical severity, variant type, age, and clinical diagnosis (i.e., male Rett syndrome or *MECP2-*related neurodevelopmental disorder) do not significantly impact the ability to respond favorably to treatment. This should be taken into consideration as clinicians screen who may benefit from treatment with trofinetide. Effectiveness across variant type would be expected since the mechanism of action of trofinetide is via the brain-derived neurotrophic factor (BDNF) pathway, and any pathogenic *MECP2* variant should alter this pathway, so treatment modifying this pathway may be effective (16, 17). Future work to include larger number of individuals with pathogenic *MECP2* variants beyond females with typical RTT should be considered or at minimum, have a side study, which includes anyone with a pathogenic or likely pathogenic *MECP2* variant.

There are several limitations to this study. First, this was a real-world, retrospective study, and there was no placebo or blinding which would be expected to result in a higher impression of effectiveness. However, the social media environment around trofinetide is mixed with many families concerned about the risk of stool changes given projected improvement. This negative impression of trofinetide could have resulted in reporting a worse outcome than observed in clinical trials. As this is an FDA- approved medication, there will not be future placebo control trials, but considering someone’s expectations of starting treatment should be considered when initiating therapy.

Second, several individuals did not complete the scheduled RSBQ assessments or missed a recommended follow-up visit leading to incomplete data sets that missed either the baseline, 3-month, or 6-month RSBQ score. We also did not limit completion of RSBQ or subjective reports of improvement to the same caregiver leading to variability in reporting. Tracking trofinetide’s efficacy and progression of improvement over a period of time without complete data sets and with caregiver variability limits the generalizability of our data. While our subjective interviews lead to variability in reporting, it did not add to caregiver burden since it was extracted from typical clinic which should be considered when discussing if trofinetide is effective and should be continued outside of clinical trials. This contrasts to the LOTUS study which required families to participate beyond what they would be asked of in clinic and still essentially collected more qualitative data (11).

Third, our population exhibited substantial variability in terms of disease severity, genetic variations, and medical co-morbidities which could have influenced individual responses to trofinetide. This differs significantly from the clinical trials, which included a much more homogeneous population. Participants with atypical Rett syndrome or *MECP2*-related neurodevelopmental disorder often present with different baseline symptoms compared to those with typical Rett syndrome, introducing additional factors to consider when assessing treatment effects across the cohort. Although our experience with these groups is small and not necessarily generalizable, a strength of this paper is demonstrating improvements across *MECP2* variants and clinical presentations suggesting future research in these populations would be beneficial to improve access to treatment and gain more data on responsiveness.

While our study has these limitations, it is the first clinic based real-world experience of trofinetide and provides clinicians with data to guide discussion with their patients to consider dosing strategies, side effect management and realistic expectations of improvement with treatment. Acknowledging that asking families to repeatedly fill out questionnaires outside of the clinic is time-consuming and recognizing that subjective caregiver reports of improvement may better reflect their perception of benefit and is less burdensome than questionnaires suggest this may be an effective way to guide discussions of continuing therapy rather than relying on RSBQ and CGI-I outside of clinical trials.

In conclusion, we present the first real world clinic-based experience using trofinetide in several *MECP2-*related disorders. We confirm the safety of this medication, redemonstrate the most common side effects, and suggest that side effects often can be managed with slowed titration, dose reductions, and medical management. We also present data which shows caregivers report improvements from trofinetide across several domains of functioning supporting the continuation of treatment independent of RSBQ score change and CGI-I. Future reports from other clinics and providers will continue to create a broader base of experience in the use of this first FDA approved medication not only in typical Rett syndrome, but across all *MECP2* - related disorders.

## Data Availability

The raw data supporting the conclusions of this article will be made available by the authors, without undue reservation.

## Footnotes

1
https://www.python.org/

2
https://posit.co/
